# The "fit but fat" concept revisited: population-based estimates using NHANES

**DOI:** 10.1186/1479-5868-7-47

**Published:** 2010-05-24

**Authors:** Glen E Duncan

**Affiliations:** 1 Department of Epidemiology, Nutritional Sciences Program, University of Washington, Seattle, WA 98195, USA

## Abstract

Low cardiovascular fitness is an independent risk factor for type 2 diabetes and cardiovascular disease in adults. The "fit but fat" concept suggests that cardiovascular fitness attenuates risk of metabolic and cardiovascular disease independent of body mass index (BMI), even among the obese. However, the proportion of U.S. adults considered both fit and obese is unknown. Thus, the purposes of this short paper were to estimate the proportion of U.S. adults who are obese yet have a high cardiovascular fitness level (fit but fat), and determine the independent effect of obesity on cardiovascular fitness. The study was a secondary data analysis of 4,675 adults (20-49 years) who completed a submaximal exercise test, from the National Health and Nutrition Examination Survey (1999-2002). Cardiovascular fitness and body weight were expressed as continuous (estimated VO_2max _[ml.kg^-1.^min^-1^] and BMI [kg/m^2^]) and categorical variables (low, moderate, and high cardiovascular fitness level; normal weight, overweight, and obese), the later using sex and age-specific criteria from the Aerobics Center Longitudinal Study and standard BMI cut-points, respectively. Using these methods, the prevalence of meeting the fit but fat definition among U.S. adults was 8.9% (95% C.I. = 6.9 - 10.9%), whereas 17.4% were overweight and high fit, and 30% were normal weight and high fit. Importantly, the proportion of low, moderate, and high cardiovascular fitness differed significantly (*p *< 0.05) by BMI level. Using multiple regression, being obese was associated with a 9.2% lower estimated VO_2max _compared to being normal weight, even after controlling for age, sex, race/ethnicity, and income. These results suggest that a small percentage of U.S. adults can be considered fit but fat, and that obesity is independently associated with reduced cardiovascular fitness. The likely explanation for the low proportion of U.S. adults who can be considered fit but fat is a low level of physical activity, which constributes to both a positive energy balance and low fitness. Thus, engaging obese adults in physical activity that is sufficient to improve cardiovascular fitness may help to reduce not only body weight, but the excess health burden in this population.

## Background

High levels of physical activity and/or cardiovascular fitness attenuate health risks associated with overweight and obesity [1-11], although it is unclear whether activity or fitness can eliminate the risks [12,13]. A common recommendation to improve both cardiovascular fitness and promote weight loss in the overweight and obese is to increase physical activity [14]. However, physical activity levels in the population are well below those recommended for health benefits [15], and rates of physician counseling about healthful lifestyles in overweight and obese patients is also typically low [16].

Given the high rates of obesity and low rates of physical activity in the population, it is unclear how many U.S. adults might be considered both fit and obese ("fit but fat"). The purpose of this short paper was to estimate the proportion of U.S. adults who are clinically obese by body mass index standards (BMI), yet have a high cardiovascular fitness level as estimated from exercise testing in a population-based study. The author hypothesized that cardiovascular fitness levels differ significantly by BMI levels, and that obesity is independently associated with reduced cardiovascular fitness.

## Methods

### Sample

The study was a secondary data analysis of 4,675 adults 20-49 years who had completed a submaximal exercise test, from the National Health and Nutrition Examination Survey (NHANES) 1999-2002. The NHANES protocol was reviewed and approved by the National Center for Health Statistic's Institutional Review Board. Written informed consent was obtained from all participants prior to testing.

### Testing

Detailed procedures for the cardiovascular fitness component of NHANES are available elsewhere [17]. Briefly, participants were screened using questionnaires and physical examination. Individuals were excluded from testing based on certain medical conditions, medication usage, and physical limitations that would interfere with cardiovascular responses to exercise. The fitness exam was performed by health technicians trained using a common protocol. The goal of each test was to elicit a heart rate that was approximately 75% of the age-predicted maximum (220-age). The test included a 2-minute warm-up, two 3-minute exercise stages, and a 2-minute cool down period. Heart rate was monitored continuously, and blood pressure was measured at the end of each stage, using an automated electronic monitor. Testing was terminated in persons who exhibited abnormal signs or symptoms (e.g., pain or pressure in the chest), or heart rate or blood pressure responses (e.g., heart rate > 85% of predicted maximal heart rate, blood pressure > 260 or 115 mmHg) during the warm-up or at the end of a stage.

### Measures

The primary outcome was estimated VO_2max _(ml.kg^-1.^min^-1^), calculated from the heart rate response to known levels of submaximal work. Using sex and age-specific criteria for adults 20-49 yr. from the Aerobics Center Longitudinal Study (ACLS), the estimated VO_2max _was also categorized as a low, moderate, or high level of cardiovascular fitness. Specifically, low cardiovascular fitness was an estimated VO_2max _below the 20^th ^percentile of the ACLS data of the same sex and age group, moderate fitness was between the 20^th ^and 59^th ^percentiles, and high fitness at or above the 60^th ^percentile.

Height was measured with a stadiometer, and weight on a self-zeroing scale with no shoes and wearing light clothing. BMI was calculated as kg/m^2 ^(continuous variable), and also expressed as a categorical variable using standard cut-points, where normal weight was BMI < 25, overweight BMI > 25 <30, and obese BMI > 30. Demographic variables were obtained by self-report.

### Analysis

Data were analyzed using SAS version 9.2 with survey software. All analyses used the four-year full sample weights (wtmec4yr) to estimate means and 95% confidence intervals (CI), and the corresponding masked variance units (pseudo-primary sampling units [SDMVPSU] and pseudo-stratum variables [SDMVSTRA]) to estimate standard errors of those means. Differences among continuous outcomes by BMI level were tested using linear regression. Prevalence and differences for cardiovascular fitness level by BMI level were compared using the Wald χ^2 ^test of association. The procedure provides design-adjusted tests of independence (no association) between row (fitness level) and column (BMI level) variables. Multiple linear regression was used to examine associations between cardiovascular fitness and BMI. Statistical significance was established at α = 0.05 *a priori*, and multiple comparisons were adjusted using the Bonferroni method. Six participants who had extreme estimated VO_2max _values (≥ 87.5 ml.min^-1.^kg^-1^, which were above the 99^th ^percentile of the sample distribution), and all participants with missing or negative sampling weights (*n *= 230), were excluded from analysis.

## Results

Select descriptive information on the sample is provided in Table 1. Using NHANES sample weights, the analytic sample size (*n *= 4,675) was eqivalent to a population-based sample size *N *= 143,225,503 subjects. Overall, 10.3 ± 1.0% (mean ± standard error) of subjects had a low cardiovascular fitness level, 33.4 ± 1.8% medium fitness, and 56.3 ± 2.1% high fitness. With respect to BMI level, overall 44.9 ± 2.0% subjects were normal weight, 34.7 ± 1.8% overweight, and 20.4 ± 1.1% obese.

**Table 1:** Select descriptive characteristics for adults 20-49 years who completed a submaximal graded exercise test in the National Health and Nutrition Examination Survey, 1999-2002.

	Mean ± SE
Age (years)	33.5 ± 0.4
Estimated VO_2max _(ml.kg^-1^.min^-1^)	41.7 ± 0.4
Body Mass Index (kg/m^2^)	26.5 ± 0.2
	**Percentage ± SE**
Sex*	
Male	54.8 ± 1.5%
Female	45.2 ± 1.5%
Race/Ethnicity*	
Mexican-American	6.4 ± 0.8%
Other Hispanic	5.9 ± 0.3%
Non-Hispanic White	76.4 ± 1.6%
Non-Hispanic Black	7.8 ± 0.9%
Other Race - Including Multi-Racial	3.5 ± 0.7%

Data presented as the mean or percent and standard error (SE). *Sex and race/ethnicity variables both sum to 100%.

Differences in major outcomes by BMI level are provided in Table 2. There was no difference (*p *> 0.05) among groups for age. However, estimated VO_2max _(ml.kg^-1.^min^-1^) was significantly higher in both normal weight and overweight groups, compared to the obese (*p *< 0.05). The distribution of cardiovascular fitness level differed significantly (*p *< 0.05) by BMI status. Overall, 8.9% of subjects were obese and had a high cardiovascular fitness level, 17.4% were overweight and high fit, and 30% were normal weight and high fit.

**Table 2:** Differences in major outcomes by body mass index level.

	Normal weight	Overweight	Obese
Age (mean years)	32.4 ± 0.8	34.4 ± 0.5	34.5 ± 0.9
Estimated VO_2max _(mean ml.kg^-1^.min^-1^)	42.9 ± 0.4*	41.8 ± 0.9*	38.7 ± 0.6
Fitness Level (percentage) †§			
Low	2.0 ± 0.5 (2,920,699)	4.3 ± 0.6 (6,188,369)	3.9 ± 0.5 (5,644,436)
Medium	12.9 ± 1.3 (18,451,215)	13.0 ± 1.1 (18,573,013)	7.6 ± 0.8 (10,819,591)
High	30.0 ± 2.3 (42,968,269)	17.4 ± 0.4 (24,868,929)	8.9 ± 1.0 (12,790,983)

Data presented as the mean or percent and standard error (SE). Symbols for statistical significance: * = different from obese at *p *< 0.05; † = distribution of fitness level different across body mass index (BMI) groups. §The nine cells for fitness level by BMI level sum to 100% and represent the distribution across the population. The weighted sample size for each cell is indicated in parenthesis for the total analytic sample *N *= 143,225,503. Fitness level was categorized using sex and age-specific criteria for adults 20-49 yr. from the Aerobics Center Longitudinal Study, where low was defined as an estimated VO_2_max below the 20^th ^percentile of the same sex and age group, moderate between the 20^th ^and 59^th ^percentiles, and high at or above the 60^th ^percentile. Cut-points used to define weight status by BMI (kg/m^2^) level are: normal weight < 25; overweight > 25 < 30; and, obese > 30.

**Figure 1:**
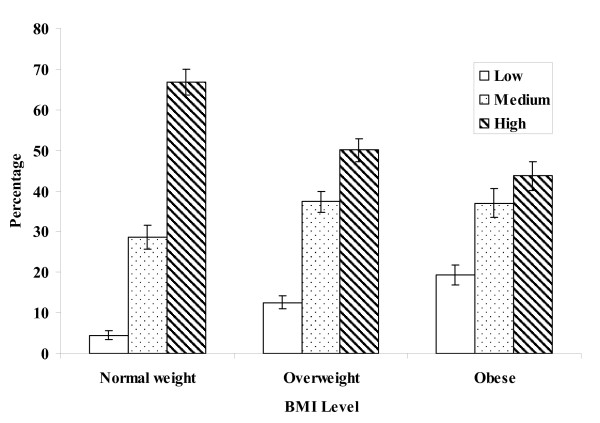
**Distribution of cardiovascular fitness level within each body mass index level**. Data presented as percentage and standard error (SE). Cardiovascular fitness level was categorized using sex and age-specific criteria for adults 20-49 yr. from the Aerobics Center Longitudinal Study, where low was defined as an estimated VO_2_max below the 20^th ^percentile of the same sex and age group, moderate between the 20^th ^and 59^th ^percentiles, and high at or above the 60^th ^percentile. Cut-points used to define weight status by body mass index (BMI, kg/m^2^) level are: normal weight <25; overweight > 25 <30; and, obese > 30.

The distribution of cardiovascular fitness levels within each BMI level is provided in Figure 1. The proportion of subjects with a high cardiovascular fitness level was over 20 percentage points lower in obese compared to normal weight adults, whereas the percentage of low cardiovascular fitness was roughly 15 percentage points higher in the obese compared to normal weight.

Regression models were constructed to examine associations between estimated VO_2max _(ml.kg^-1.^min^-1^) and BMI, which was entered both as a continuous and categorical variable. In the best fitting model (*r*^2 ^= 0.263), using categorical BMI, being obese was associated with a 9.2% and overweight a 6.1% lower estimated VO_2max_, compared to being normal weight, controlling for age, sex, race/ethnicity, and income (both *p *< 0.05).

## Discussion

The fit but fat concept suggests that high levels of cardiovascular fitness attenuate or potentially eliminate risks associated with several metabolic and cardiovascular disease outcomes independent of BMI, even among individuals who are obese. This study demonstrates that a relatively small percentage of U.S. adults, about 9%, can be considered fit but fat. Furthermore, obesity is independently associated with reduced cardiovascular fitness at the population level. In contrast, about 17% of U.S. adults completing the fitness-testing component of NHANES were overweight and had a high cardiovascular fitness level, while 30% were normal weight and high fit.

The distribution of cardiovascular fitness levels among U.S. adults differed significantly by BMI level. Both sex and race/ethnicity also differed significantly by BMI level (data not shown). Of note, there was a higher relative proportion of Non-Hispanic Black and lower proportion of Non-Hispanic White adults in the obese group. This difference may have contributed to the difference found in the distribution of high cardiovascular fitness levels across BMI levels. This is based on reports that Non-Hispanic Black adults, particularly women, have lower cardiovascular fitness levels than do other major race/ethnicity groups tested in the NHANES sample [18,19].

Although the percentage of adults who were fit but fat as defined in this study was relatively small across BMI levels in the population (Table 2), a different picture emerges when examining cardiovascular fitness levels within each BMI level. Among obese adults, about 20% had a low cardiovascular fitness level whereas 80% achieved a medium or high level (Figure 1). Among the overweight, 12.5% had low cardiovascular fitness and 87.5% had medium or high fitness. These findings are somewhat encouraging because they demonstrate that overweight and obese individuals can achieve a medium to high cardiovascular fitness level, which could potentially mitigate some of the deleterious effects of excess body weight on health.

Of course, not all studies demonstrate that high levels of cardiovascular fitness attenuate health risks independently of excess body weight, perhaps refuting the fit but fat concept. For example, Stevens and colleagues [9] concluded that although both fitness and fatness were risk factors for mortality, being fit did not completely reverse the increased risk associated with excess adiposity. Christou et al. [20] found that body fatness was a better predictor of cardiovascular disease risk profile than aerobic fitness in healthy men. Finally, in the Quebec Family Study [21], the effects of physical fitness on individual components of the metabolic syndrome were attenuated after considering total and abdominal adiposity.

Similarly, several studies have demonstrated that BMI may be more important than is physical activity in predicting development of adverse health outcomes [22-24]. However, it is difficult to compare "head to head" results of various studies examining the relationships among physical activity and/or physical fitness and BMI and/or adiposity with health because these measures describe different concepts and are therefore not interchangeable. Indeed, as Blair and colleagues suggest in a comprehensive review [25], it is not possible at this time to conclude whether activity or fitness is more important for health.

Several limitations are noteworthy. First, NHANES is a cross-sectional survey, which negates drawing causal inferences as to the underlying relationship between cardiovascular fitness and obesity. Related, BMI is an imperfect surrogate measure of adiposity and the potential for misclassification has been documented [26,27]. There is also potential for misclassification of fitness level using an estimate of cardiovascular fitness from a submaximal instead of a maximal, graded exercise test. Differences in the proportions of low, moderate, and high cardiovascular fitness among BMI levels may have been caused by a potential for bias or misclassification because the cut-points used to define these levels were derived from the ACLS, which is a mostly well-educated, white sample. Finally, the distribution of cardiovascular fitness levels by BMI levels among U.S. adults provided in this report may not reflect the actual distribution in the population because of the select age and physical status of the sample completing exercise testing in NHANES.

Despite the limitations noted, this study demonstrates that few U.S. adults are both highly fit and obese across the population. However, the data also demonstrate that obese and overweight adults can achieve a moderate to high level of cardiovascular fitness. The 20% or so most unfit overweight and obese adults are therefore optimal targets for intervention. Because the common recommendation to improve both cardiovascular fitness and promote weight loss in the overweight and obese is to increase physical activity [14], the findings from this report might better serve to suggest that obese and overweight individuals not only move around more (i.e., increase habitual physical activity), but that they move around more at a level that is sufficient to improve cardiovascular fitness (i.e., activity of a sufficient dose). Although changes in cardiovascular fitness in response to training are variable and determined in part by genetic and common environmental factors [28-30], even small increases in activity and/or fitness may provide health benefits.

## Competing interests

The author declares that they have no competing interests.

## Authors' contributions

GD conceived the study, performed the statistical analyses, and wrote the manuscript.
